# Supplementary material for: Angelica sinensis polysaccharide as potential protectants against recurrent spontaneous abortion: focus on autophagy regulation
Source: Front Med (Lausanne). 2025 Jan 15;12:1522503. doi: 10.3389/fmed.2025.1522503 (PMC11774876; doi:10.3389/fmed.2025.1522503)
Supplement: Supplementary material S1 — The main instruments used during the LC-MS process, along with their models/specifications and manufacturers. [file Supplementary_file_1.zip › Metabolomics sequencing data FC1.2/8.代谢通路分析/Exposure_Ctrl/all/KEGG/mmu05133.html]

KEGG PATHWAY: Pertussis - Mus musculus (house mouse)


# Pertussis - Mus musculus (house mouse)


[
Pathway menu
| Organism menu
| Pathway entry
| Show description
| Download
| Help
]

Pertussis, also known as whooping cough, is an acute respiratory infectious disease caused by a bacteria called Bordetella Pertussis. The characteristic symptoms are paroxysmal cough, inspiratory wheezing and post-tussive vomiting.
Following the inhalation of respiratory secretions from an infected individual, bacteria enter the upper respiratory tract and adhere to epithelial cells. Several adhesion factors have been implicated: the filamentous hemagglutinin (FHA), fimbriae, and pertactin (Prn).
Pertussis toxin (Ptx) and adenylate cyclase toxin (ACT) have been identified so far as major protein toxins of B. pertussis. PTX is a hexameric AB5-type exotoxin. Catalytic A subunit catalyzes the ADP-ribosylation of the Gi subunits of the heterotrimeric G protein, then inhibits multiple downstream pathways. ACT is able to penetrate the cytoplasmic membrane of host cells and becomes activated through the cleavage and the binding of calmodulin (CaM). Activated ACT converts ATP to cyclic AMP and subverts cellular signaling pathways.


##### Option

Scale:


100%

Image resolution:


 High

##### Search

##### ID search

##### Color


KGML

Image (png) file 1x
